# Draft genome of an NDM-5-producing *Huaxiibacter chinensis* isolated from a urinary tract infection

**DOI:** 10.1128/mra.00847-26

**Published:** 2026-09-08

**Authors:** Yu Feng, Meiyun Zong

**Affiliations:** 1 Laboratory of Pathogen Research, West China Hospital, Sichuan University 34753, Chengdu, China; 2 Population Health Sciences Undergraduate Program, Institute of Epidemiology and Health Care, University College London 384711, London, United Kingdom; University of Pittsburgh School of Medicine, Pittsburgh, Pennsylvania, USA

**Keywords:** NDM-5, urinary tract infection, *Huaxiibacter chinensis*

## Abstract

*Huaxiibacter chinensis* is a recently proposed Enterobacteriaceae species with few public genomes. We report the draft genome of urinary isolate 195099, carrying multiple resistance genes, including *bla*_NDM-5_, documenting a carbapenem-resistant clinical isolate of this uncommon species.

## ANNOUNCEMENT

Antimicrobial resistance remains a major global threat, particularly carbapenemase-producing Enterobacteriaceae (1). *Huaxiibacter chinensis* and the genus *Huaxiibacter* were proposed in 2022 from a human sputum isolate (2). Reports from mink pneumonia (3) and hibernating bats (4) suggest diverse host-associated niches, but genomic data remain limited, with only six assemblies available in GenBank. Here, we report a clinical *H. chinensis* genome from a human urine isolate.

Strain 195099 was recovered in 2023 from the urine of a patient with a urinary tract infection after radical cystectomy at our hospital by aerobic overnight culture on LB agar (Hopebio, Qingdao, China) at 37°C. The isolate was initially assigned to the *Enterobacter cloacae* complex by MALDI-TOF MS (Bruker Daltonics, Bremen, Germany), and antimicrobial susceptibility was tested using the Vitek II system (bioMérieux, Marcy-l’Étoile, France). A single colony was grown overnight in LB broth at 37°C, and genomic DNA was extracted using QIAamp DNA Blood Mini Kit (Qiagen, Hilden, Germany). Libraries were prepared from 350-bp sheared DNA fragments using the NEBNext Ultra II DNA Library Prep Kit for Illumina (New England Biolabs, Ipswich, MA, USA) according to the manufacturer’s protocol without modification. NovaSeq 6000 (Illumina, San Diego, CA, USA) sequencing generated 4,377,249 150-bp paired-end read pairs comprising 1.31 Gb, or approximately 262× coverage. Reads were quality controlled using fastp v1.3.6 (5) and assembled *de novo* with SPAdes v4.2.0 in isolate mode (6). Species identification was determined by average nucleotide identity (ANI) analysis against genomes of Enterobacteriaceae type strains using skani v0.3.2 (7). Antimicrobial resistance genes were detected with AMRFinderPlus v4.2.7 (8). Whole-genome single-nucleotide polymorphism (SNP) analysis was performed using Snippy v4.6.0 (https://github.com/tseemann/snippy), followed by IQ-TREE v3.1.2 (9) inference under GTR + F + ASC + G4 model with 1,000 ultrafast bootstraps. Default settings were used unless otherwise specified.

The draft genome comprised 57 contigs totaling 5,010,389 bp, with an *N*_50_ of 387,278 bp and a G + C content of 53.98%. NCBI quality assessment and PGAP v6.11 (10) annotation indicated 100% completeness and identified 4,692 coding genes, 108 RNA genes, and 56 pseudogenes. ANI analysis showed 99.84% identity to the type strain of *H. chinensis* (GCA_022171985.1), reclassifying strain 195099 as *H. chinensis*. The resistome included *bla*_NDM-5_, *bla*_OXA-10_, *aadA1*, *ant(2″)-Ia*, *catA1*, *catB8*, *oqxA*, *oqxB*, *sul1*, and *ble*, consistent with resistance to amoxicillin-clavulanic acid (MIC ≥ 32 µg/mL), ampicillin-sulbactam (≥32 µg/mL), ceftazidime (≥64 µg/mL), cefepime (16 μg/mL), doripenem (≥8 µg/mL), imipenem (≥16 µg/mL), meropenem (≥16 µg/mL), ciprofloxacin (≥4 µg/mL), and levofloxacin (4 μg/mL), while remaining susceptible to aztreonam (≤1 µg/mL) and tigecycline (≤0.5 µg/mL).

**Fig 1:**
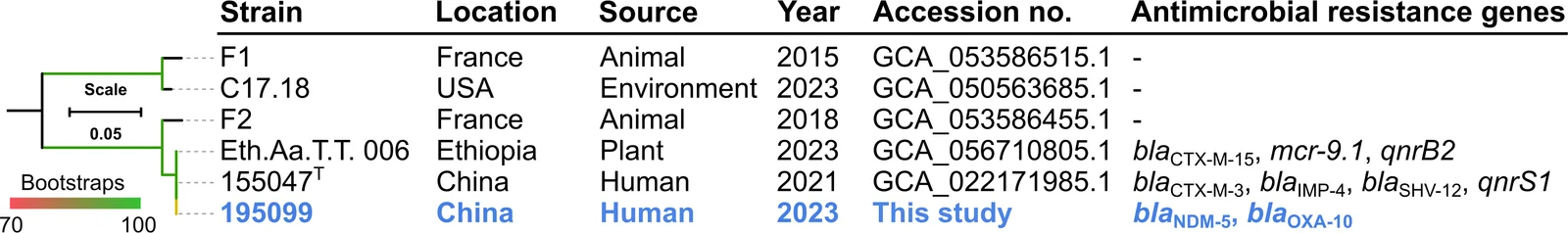
Whole-genome SNP phylogeny of *Huaxiibacter chinensis* strain 195099 and five public pure-culture genomes. The metagenome-derived assembly (GenBank accession GCA_052110535.1) was excluded from the analysis. Metadata include strain identifier, year of isolation, geographic origin, isolation source, assembly accession, and major antimicrobial resistance genes associated with resistance to carbapenems, cephalosporins, quinolones, and colistin. Branch color represents bootstrap support values, ranging from 70 (red) to 100 (green). The scale bar indicates the number of substitutions per site. Strain Eth.Aa.T.T. 006 was assigned to a plant source based on its BioSample description, which states that it was isolated from the Ethiopian medicinal plant *Artemisia abyssinicus*, although no related publication was identified.

Whole-genome SNP-based phylogenetic analysis of strain 195099 and the five pure-culture *H. chinensis* genomes showed broad geographic, source, and genetic diversity (Fig. 1). Strain 195099 was closest to an Ethiopian isolate and the type strain, differing by 857 and 928 SNPs, respectively, while distances to the remaining genomes exceeded 30,000 SNPs. These findings document a carbapenem-resistant clinical urine isolate of *H. chinensis* and highlight this uncommon species as a potential reservoir of clinically important resistance genes.

## Data Availability

The genome assembly has been deposited in GenBank under accession number JCAOGF000000000. The corresponding raw sequencing reads have been deposited in the Sequence Read Archive under accession number SRR39467693.
